# Assignment of Serotype-Specific IgG1, IgG2, and IgA Weight-Based Antibody Units to the Human Pneumococcal Standard Reference Serum, 007sp

**DOI:** 10.1128/mSphere.00400-19

**Published:** 2019-06-19

**Authors:** Scott Jones, Kier Finnegan, Jae Hee Wee, Polly D’Argoeuves, Lucy Roalfe, Suzanne Byrne, Sarah Heffernan, Adam Hunt, Marina Johnson, Tessa Jones, Emma Pearce, Hayley Richardson, Vasili Thalasselis, David Goldblatt

**Affiliations:** a Department of Immunobiology, UCL Great Ormond Street Institute of Child Health, NIHR Biomedical Research Centre, London, United Kingdom; University of Maryland School of Medicine

**Keywords:** 007sp, ELISA, Gram-positive bacteria, IgA, IgG1, IgG2, *Streptococcus pneumoniae*, antibody standard, immunization, immunoserology, lot 89SF, serology, vaccines

## Abstract

In 2011, the human pneumococcal standard reference serum, 007sp, was established as a replacement for the previous standard, lot 89SF, supplies of which were dwindling. The pneumococcal reference serum is used primarily in the standardized pneumococcal enzyme-linked immunosorbent assay (World Health Organization reference enzyme-linked immunosorbent assay) but has also been used in functional assays. Serotype-specific IgG values for 24 pneumococcal capsular serotypes have previously been assigned to 007sp by bridging to the original values derived for lot 89SF. In this study, by bridging to existing values in lot 89SF, we assign weight-based serotype-specific IgA, IgG1, and IgG2 to 007sp for 11 pneumococcal capsular serotypes (1, 3, 4, 5, 6B, 7F, 9V, 14, 18C, 19F, and 23F), as well as serotype 19A-specific IgA. Concentrations for serotype-specific IgA, IgG1, and IgG2 present in 007sp were comparable to those previously assigned to lot 89SF. In addition, the concentration of serotype-specific IgG1 plus IgG2 assigned to 007sp significantly correlated to previously assigned 007sp IgG values. The accuracy of antibody assignments to 007sp from lot 89SF was assessed by comparing the concentration of serotype-specific IgA, IgG1, and IgG2 in 16 unknown samples using both 007sp and lot 89SF as the standard. Interpolated values for the unknown samples were highly correlated with average *R*^2^ values of 0.9729, 0.9951, and 0.9933 for IgA, IgG1, and IgG2, respectively, for all serotypes demonstrating the precise nature assignments to 007sp made in this study. Nonparallelism between 007sp and lot 89SF has precluded the derivation of serotype-specific IgM values.

**IMPORTANCE** A well-characterized antibody standard is an indispensable reagent for use in assays designed to measure antibodies with precision and where assays between laboratories need to be comparable. The human pneumococcal standard reference serum, lot 89SF, greatly facilitated the standardization of enzyme-linked immunosorbent assay methodologies during a critical period when the first pneumococcal polysaccharide-conjugate vaccines were being evaluated for licensure. Due to dwindling supplies of lot 89SF, a new reference standard, 007sp, was produced in 2011. Understanding the isotype and subclass composition of either natural or vaccine induced responses to pathogens has assumed increasing importance. In this study, we have assigned IgA, IgG1, and IgG2 values to pneumococcal serotypes 1, 3, 4, 5, 6B, 7F, 9V, 14, 18C, 19A, 19F, and 23F by bridging to existing values in lot 89SF.

## Abstract (precis)

A well-characterized antibody standard is an indispensable reagent for use in assays designed to measure antibodies with precision and where assays between laboratories need to be comparable. The human pneumococcal standard reference serum, lot 89SF, greatly facilitated the standardization of enzyme-linked immunosorbent assay methodologies during a critical period when the first pneumococcal polysaccharide-conjugate vaccines were being evaluated for licensure. Due to dwindling supplies of lot 89SF, a new reference standard, 007sp, was produced in 2011. Understanding the isotype and subclass composition of either natural or vaccine induced responses to pathogens has assumed increasing importance. In this study, we have assigned IgA, IgG1, and IgG2 values to pneumococcal serotypes 1, 3, 4, 5, 6B, 7F, 9V, 14, 18C, 19A, 19F, and 23F by bridging to existing values in lot 89SF.

## INTRODUCTION

The human pneumococcal standard reference serum, lot 89SF, was a critical regent during the standardization of enzyme-linked immunosorbent assay (ELISA) methodologies at a critical period when the first pneumococcal polysaccharide-conjugate vaccines were being evaluated for licensure. Lot 89SF was used in serotype-specific ELISAs designed to measure IgG antibody specific for individual pneumococcal capsular polysaccharides. Serotype-specific weight-based values for IgA, IgG, and IgM in lot 89SF were originally derived for serotypes 1, 3, 4, 5, 6B, 7F, 9V, 14, 18C, 19F, and 23F by Quataert et al. (1) and then extended to cover all serotypes in the 23-valent pneumococcal polysaccharide vaccine (2). IgG1 and IgG2 serotype-specific values for serotypes 3, 6B, 14, 19F, and 23F were assigned to lot 89SF in 1998 (3) and to serotypes 1, 4, 5, 7F, 9V, and 18C in 2005 (3, 4). Due to dwindling supplies of lot 89SF, a new reference standard, 007sp, was developed in 2011 (5). This serum was generated under an U.S. Food and Drug Administration (FDA)-approved clinical protocol, in which 278 adult volunteers were immunized with the 23-valent unconjugated polysaccharide vaccine, Pneumovax II. To date, serotype-specific IgG concentrations have been assigned to 007sp for the 23 pneumococcal capsular serotypes that are in the 23-valent capsular polysaccharide vaccine and serotype 6A (5–7). Values for IgA, IgM, and IgG subclasses have not yet been assigned to 007sp.

Pneumococcal vaccination induces IgG, IgA, IgM, IgG1, and IgG2 responses. The dominant isotype and serotype composition induced by vaccination varies depending on type of vaccine used (pure polysaccharide versus conjugate) and with age (1, 8). While circulating IgG has been correlated with serotype-specific protection against invasive pneumococcal disease (IPD), there is still considerable uncertainty about the exact effector mechanism(s) that mediate protection both against IPD and other infection syndromes and nasopharyngeal carriage. To this end, it remains relevant to have the appropriate reagents in place to estimate all serotype-specific isotypes and IgG1 and IgG2 in the context of vaccination and natural immunity. Furthermore, variable contribution of the isotypes induced by vaccination to functional immune responses emphasize the relevance of being able to estimate serotype-specific antibodies other than IgG (9, 10).

In this study, we assign preliminary serotype-specific concentrations of IgA, IgG1, and IgG2 to the human pneumococcal standard reference serum, 007sp. The accuracy of antibody assignments to 007sp bridged from lot 89SF was then assessed by comparing the concentration of serotype-specific IgG1, IgG2, and IgA in 16 “unknown” samples using previously assigned antibody values to lot 89SF and antibody values assigned to 007sp in this study.

## RESULTS

**FIG 1:**
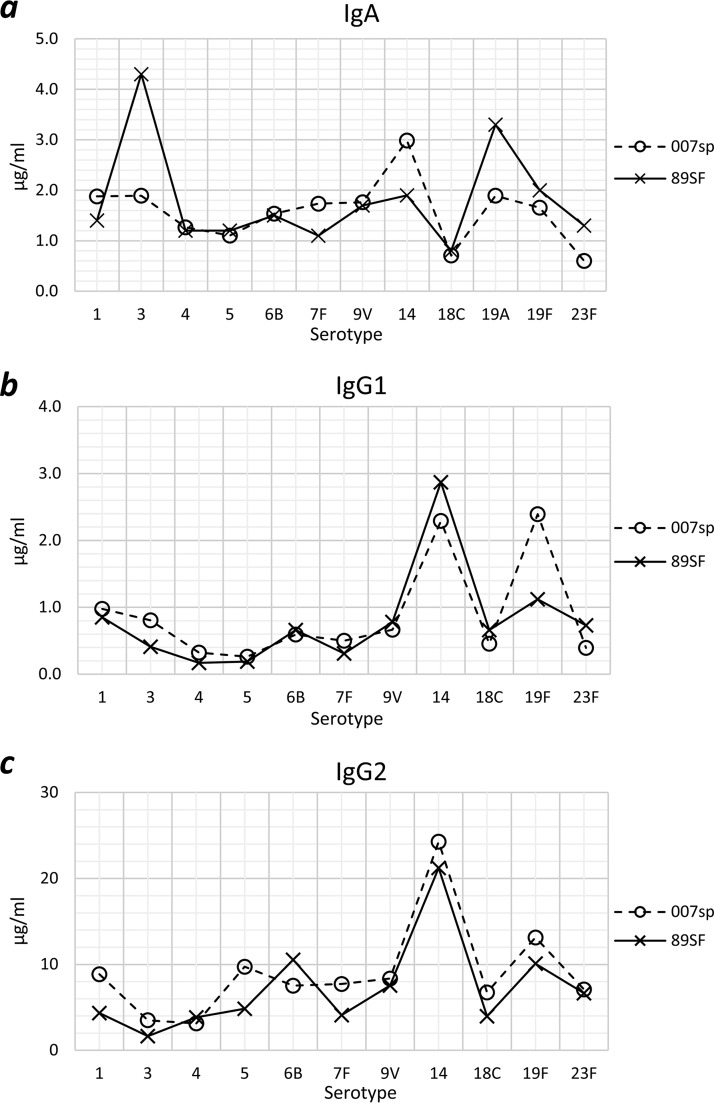
Comparison of the original assigned values for serotype-specific IgA (a), IgG1 (b), and IgG2 (c) antibody in lot 89SF and those assigned to 007sp in this study. Each point represents the geometric mean concentrations of serotype-specific antibody assigned to lot 89SF (crosses; solid line) and 007sp (open circles, dashed line).

**FIG 2:**
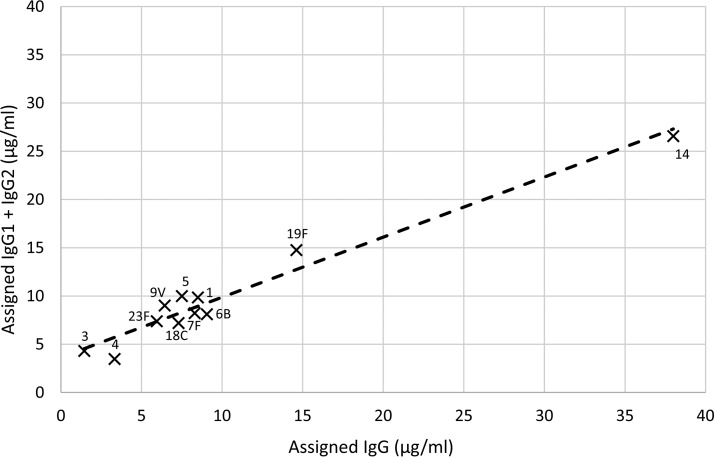
Scatter plot showing the correlation (linear regression; dotted line) between previously assigned serotype-specific IgG to 007sp and the sum of serotype-specific IgG1 and IgG2 to 007sp assigned in this study. Each point represents geometric mean antibody concentrations for one pneumococcal capsular serotype (individually labeled). *R*^2^ = 0.9546; *P* = 2e–7; *n* = 11 serotypes.

The consistency of antibody concentrations assigned to 007sp in this study was assessed by calculating the coefficient of variation (CV) between repeats for each serotype. Repeats were highly consistent with CVs of <18% for IgA, IgG1, and IgG2. Analysis of variance (ANOVA) models were used to estimate IgA, IgG1, and IgG2 antibody concentrations for each of the serotypes in 007sp. Values and 95% confidence intervals (CIs) were obtained by back-transforming the estimated log-transformed concentrations and associated 95% CIs. Concentrations of IgA, IgG1, and IgG2 assigned to 007sp for each serotype are shown in Table 1. These values were derived by the double absorption of 007sp using pneumococcal cell wall polysaccharide (CWPS) and purified serotype 22F polysaccharide and thus in future both standard and unknown tests samples should be doubly adsorbed. Comparisons of IgA, IgG1, and IgG2 values assigned to 007sp and the original values for lot 89SF are shown in Fig. 1. The correlation of previously assigned serotype-specific IgG and IgG1 + 2 values assigned to 007sp in this study was highly significant with an *R*^2^ value of 0.9546, despite the fact that the assays use different secondary antibodies of undetermined affinities and are thus not optimized for comparability to each other (Fig. 2). When assessing the relative proportion of IgG1 and IgG2 binding to individual serotypes, for most serotypes the response was dominated by IgG2 (average G1/G2 ratio, 1:12.9), which was similar to the ratio seen in lot 89SF (1:10.9). The only exception was serotype 3-specific IgG1, which represented approximately 19% of the IgG response and was similar in 007sp to that observed in lot 89SF (3).

**TABLE 1:** Geometric mean concentrations of serotype-specific IgA, IgG1, and IgG2 (μg/ml) in 007sp assigned by reference to lot 89SF^*a*^

Serotype	IgA			IgG1			IgG2		
	GMC (μg/ml)	95% CI	*n*	GMC (μg/ml)	95% CI	*n*	GMC (μg/ml)	95% CI	*n*
1	1.879	1.86–1.91	40	0.976	0.90–1.537	20	8.866	7.133–11.019	20
3	1.893	1.83–1.98	40	0.807	0.73–1.199	20	3.503	3.166–3.877	20
4	1.266	1.24–1.29	40	0.325	0.30–0.511	19	3.142	1.967–5.021	20
5	1.103	1.08–1.12	40	0.262	0.24–0.388	20	9.731	6.129–15.45	20
6B	1.536	1.52–1.55	40	0.592	0.55–0.845	25	7.527	5.698–9.943	20
7F	1.734	1.70–1.77	40	0.500	0.42–1.617	18	7.726	5.657–10.552	20
9V	1.763	1.74–1.79	40	0.665	0.64–0.806	20	8.355	6.791–10.281	20
14	2.984	2.85–3.12	40	2.291	2.19–2.9	20	24.286	19.951–29.563	20
18C	0.708	0.693–0.723	40	0.456	0.41–0.869	19	6.749	4.277–10.65	20
19A	1.892	1.86–1.92	40						
19F	1.655	1.62–1.69	40	1.623	1.51–2.393	20	13.142	8.373–20.626	20
23F	0.600	0.591–0.609	40	0.304	0.29–0.395	20	7.088	6.201–8.101	20

a The geometric mean concentration (GMC), 95% confidence interval, and number of replicates (*n*) for each assignment are shown.

**FIG 3:**
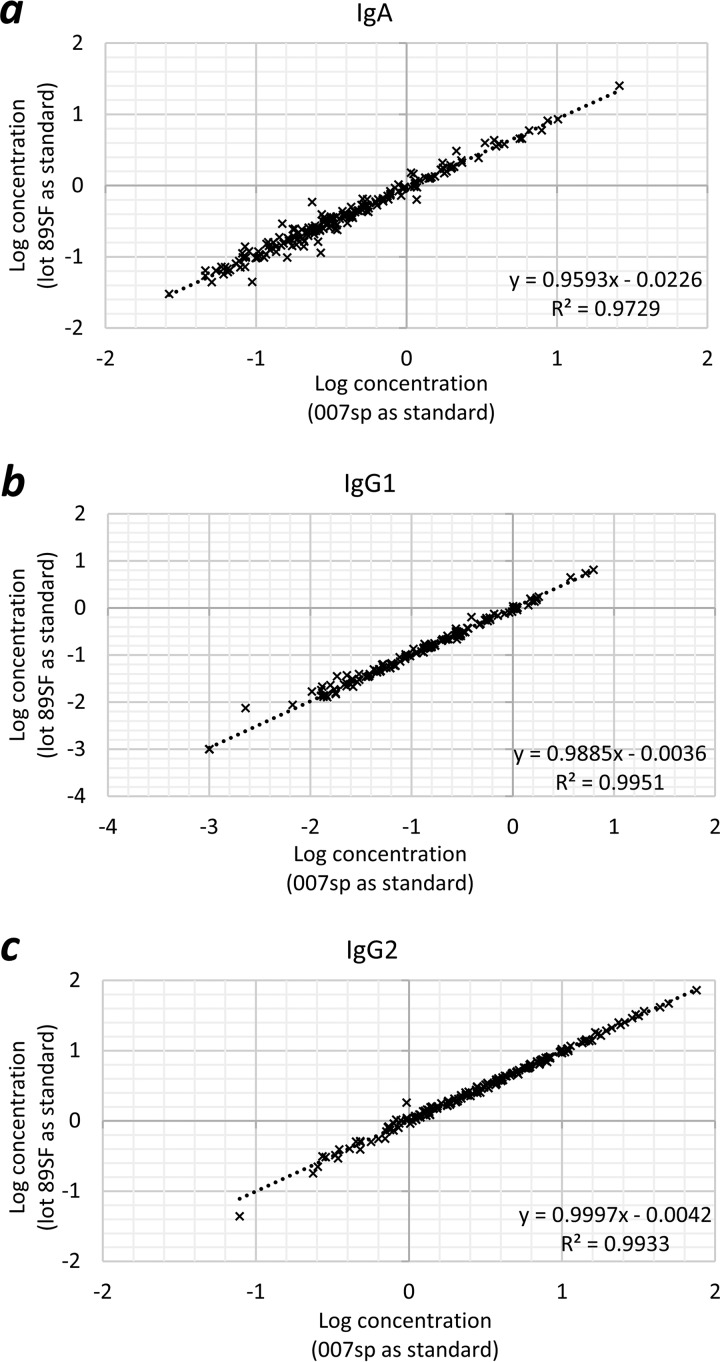
Correlation between serotype-specific IgA (a), IgG1 (b), and IgG2 (c) log concentrations interpolated using lot 89SF (*y* axis) and 007sp (*x* axis) as the standard is shown (*n* = 16 samples run three times each). The linear equations and *R*^2^ values for each assay are included. Each point (crosses) represents the average log concentration for each sample after three experiments (*n* = 11 serotypes).

The accuracy of antibody assignments to 007sp bridged from lot 89SF was assessed by measuring the concentration of serotype-specific IgG1, IgG2, and IgA in 16 “unknown” samples (FDA opsonophagocytic assay [OPA] calibration serum panel). Lot 89SF and 007sp were used independently as the antibody standards, and the interpolated serotype-specific antibody values were compared for each of the 16 unknown samples. Scatter plots showing the correlation between the concentrations of serotype-specific IgA, IgG1, and IgG2 in the panel of 16 sera using lot 89SF and 007sp for each antibody are shown in Fig. 3. Coefficients of variation between values interpolated using lot 89SF and 007sp for all serotypes were <20%. The correlation (*R*^2^ values) between values interpolated using lot 89SF and 007sp for all serotypes were ≥0.95. The fold differences between values interpolated using lot 89SF and 007sp for all serotypes were between 0.9 and 1.2. The geometric mean concentrations of each “unknown” sample tested are detailed in Table S1 in the supplemental material.

10.1128/mSphere.00400-19.1 TABLE S1 Geometric mean concentrations (*n* = 6) of serotype-specific IgA, IgG1, and IgG2 (μg/ml) in 16 “unknown” samples measured using both 007sp and lot 89SF as the assay standards. Download Table S1, DOCX file, 0.02 MB. © Crown copyright 2019. 2019 Crown This content is distributed under the terms of the Creative Commons Attribution 4.0 International license.

## DISCUSSION

A well-characterized antibody standard is an indispensable reagent for use in assays designed to measure antibodies with precision and where assays between laboratories need to be comparable. Understanding the isotype and subclass composition of either natural or vaccine induced responses to pathogens has also assumed increasing importance, particularly for pathogens that are found on mucosae such as in the nasopharynx. 007sp was established when stocks of the existing internationally accepted human pneumococcal standard reference serum, lot 89SF, were depleted. After manufacturing 007sp, initial efforts focused on assigning serotype-specific IgG levels from those assigned to lot 89SF. With that complete, we have assigned to 007sp weight-based serotype-specific IgA, IgG1, and IgG2 for 12 pneumococcal serotypes to aid in the evaluation of the contribution of isotype and subclass to responses to pneumococcal infection and vaccines. 19A assignment was only possible for IgA since IgG1 and IgG2 antibody levels to 19A were never assigned to lot 89SF.

As expected, the concentration of serotype-specific IgA, IgG1, and IgG2 present in 007sp were generally similar to those previously assigned to lot 89SF since both standards were manufactured from the sera of adults immunized with pneumococcal polysaccharide vaccine. The exceptions to this were the levels of serotype 3- and 19A-specific IgA, which were higher in lot 89SF than in 007sp, and IgG1 specific for 19F, which was higher in 007sp than in lot 89SF. These two standard sera were established over a period of time separated by decades. Natural exposure to pneumococcal serotypes in the United States cohorts immunized with the pneumococcal polysaccharide vaccine to prepare 89SF and 007sp, respectively, will have been very different since 89SF was made in the pre-PCV era, while 007sp was made in the United States in 2009/10 after 10 years of PCV use in infants. IgG2 dominated the response to all serotypes with the highest relative contribution being to serotype 5 (97%).

The accuracy of antibody assignments to 007sp bridged from lot 89SF was assessed by comparing the concentration of serotype-specific IgG1, IgG2, and IgA in 16 unknown samples using both 007sp and lot 89SF as the standard. Interpolated values for the unknown samples were highly correlated, with average *R*^2^ values of 0.9729, 0.9951, and 0.9933 for IgA, IgG1, and IgG2, respectively, for all serotypes. The fold differences between the interpolated antibody values for the 16 unknown samples using both lot 89SF and 007sp were minimal and calculated to be between 0.9 and 1.2 for all serotypes. Finally, the coefficients of variation between interpolated antibody values were small and were calculated as <20% for all serotypes. These comparisons go some way into proving the precise nature of the serotype-specific IgA, IgG1, and IgG2 assignments to 007sp made in this study.

Serotype-specific IgA antibody units remain to be assigned for 12 additional pneumococcal serotypes assigned for lot 89SF. Nonparallelism between 007sp and lot 89SF has thus far precluded the derivation of IgM-specific values for 007sp. In addition, further work assigning serotype-specific IgM, IgA, IgG1, and IgG2 values to a panel of World Health Organization (WHO) quality control sera for these capsular serotypes will be undertaken in due course (5, 6). Previous weight-based serotype-specific antibody value assignment studies have been collaborative efforts combining data from multiple laboratories (5, 6). Antibody values assigned to 007sp in this study are based on the data from one laboratory, and verification by other laboratories would be important.

## MATERIALS AND METHODS

### Collection of human sera.

The human pneumococcal standard reference serum, 007sp, was developed as previously described (5). Sera from 16 donors who took part on the development of 007sp were stored individually to act as new calibration sera (FDA OPA calibration sera) (11). Sera was found to be free of HIV and hepatitis B and hepatitis C.

### Laboratory methods.

The concentrations of serotype-specific IgG1, IgG2, and IgA antibody present in 007sp were inferred from the standard reference serum, lot 89SF, using the standardized pneumococcal reference ELISA as previously described (12). Briefly, 96-well flat-bottomed microtiter plates were coated with capsular polysaccharide antigens (ATCC; LGC Standards, Teddington, UK) from pneumococcal serotypes 1, 3, 4, 5, 6B, 7F, 9V, 14, 18C, 19A, 19F, and 23F. Both 89SF and 007sp were double absorbed with CWPS and purified serotype 22F polysaccharide to neutralize the anti-cell wall polysaccharide and nonspecific homologous antibodies to serotype 22F, as described in the WHO reference ELISA protocol (13). Plates were washed, and a titration of the 007sp and lot 89SF was added. The unknown (007sp) was run in duplicate through each ELISA a minimum of 18 times. Plates were incubated and washed again, and prediluted alkaline phosphatase-conjugated goat anti-human IgA (Sigma-Aldrich Company, Ltd.), alkaline phosphatase-conjugated mouse anti-human IgG1 (Abcam, Cambridge, UK), or alkaline phosphatase-conjugated mouse anti-human IgG2 (Source BioScience, Nottingham, UK) was added to each well. After a final incubation, the plates were washed a final time and *p*-nitrophenyl phosphate substrate (Sigma-Aldrich Company, Ltd.) was added. The reaction was stopped by adding 3 M NaOH (Thermo Fisher Scientific Oxoid, Ltd.) to each well. Plates were read using a microtiter plate reader (SPECTROstar Omega; BMG Labtech, Ltd., Buckinghamshire, UK) at 405 and 690 nm.

In order to assess the accuracy of antibody assignments to 007sp, the concentration of serotype-specific IgG1, IgG2, and IgA antibody in a panel of OPA calibration serum was measured using both antibody standards and compared. A panel of 16 sera was run with both antibody standards three times each, as described above.

### Statistical analysis.

For the assignment of values to 007sp, there were between 18 and 40 determinations of IgA, IgG1, and IgG2, respectively, for each capsular serotype. Serotype-specific IgA, IgG1, and IgG2 values were estimated for each serotype with a linear-random-effects ANOVA model. All models were fitted independently of serotype, and 95% CIs were estimated by serotype. Data were analyzed after log transformation of ELISA IgA, IgG1, and IgG2 concentrations. The geometric mean of the log concentrations for each serotype was calculated using lot 89SF antibody concentrations and estimated using ANOVA models obtained by back-transforming the estimated log-transformed concentrations and associated 95% CIs. Studentized residual plots were used to detect any outliers, which were removed from the analysis of the final assigned concentrations. All statistical analyses were performed using Excel (version 16.0.4639.1000) for Windows.

Mean serotype-specific IgG1, IgG2, and IgA antibody values assigned to the FDA OPA calibration sera panel, using both lot 89SF and 007sp antibody standards, were compared by calculating the coefficient of variation, the fold difference, and the correlation between interpolated antibody values for each serotype (*n* = 16 samples).
