# Supplementary material for: Assignment of Serotype-Specific IgG1, IgG2, and IgA Weight-Based Antibody Units to the Human Pneumococcal Standard Reference Serum, 007sp
Source: mSphere. 2019 Jun 19;4(3):e00400-19. doi: 10.1128/mSphere.00400-19 (PMC6584377; doi:10.1128/mSphere.00400-19)
Supplement: TABLE S1 [file mSphere.00400-19-st001.docx]

| Serotype | 1 | 3 | 4 | 5 | 6B | 7F | 9V | 14 | 18C | 19A | 19F | 23F |
| --- | --- | --- | --- | --- | --- | --- | --- | --- | --- | --- | --- | --- |
| *Sample Nō* | *IgA (µg/ml)* | | | | | | | | | | | |
| 714 | 0.090 | 0.174 | 0.105 | 0.111 | 0.135 | 0.066 | 0.113 | 0.210 | 0.059 | 0.391 | 0.221 | 0.066 |
| 722 | 0.130 | 0.291 | 0.112 | 0.166 | 0.343 | 0.075 | 0.184 | 0.386 | 0.132 | 0.187 | 0.165 | 0.145 |
| 726 | 0.066 | 0.129 | 0.206 | 0.098 | 0.097 | 0.048 | 0.077 | 0.124 | 0.050 | 0.293 | 0.541 | 0.090 |
| 732 | 0.104 | 0.069 | 0.055 | 0.059 | 0.067 | 0.063 | 0.078 | 0.098 | 0.059 | 0.092 | 0.192 | 0.028 |
| 736 | 2.286 | 0.596 | 0.153 | 0.247 | 0.141 | 0.644 | 1.074 | 0.334 | 0.463 | 0.332 | 0.384 | 0.082 |
| 744 | 1.681 | 0.960 | 1.768 | 0.312 | 0.945 | 0.220 | 2.730 | 3.661 | 0.682 | 8.433 | 0.312 | 0.224 |
| 746 | 0.385 | 0.582 | 0.327 | 0.297 | 0.465 | 0.302 | 0.517 | 0.407 | 0.100 | 0.808 | 0.774 | 0.317 |
| 750 | 0.301 | 0.341 | 0.519 | 0.240 | 0.215 | 0.183 | 0.483 | 4.093 | 0.146 | 0.967 | 0.326 | 1.292 |
| 752 | 0.664 | 1.911 | 1.957 | 5.194 | 9.365 | 1.860 | 5.111 | 6.940 | 1.903 | 25.591 | 2.226 | 3.988 |
| 754 | 1.948 | 1.288 | 0.756 | 0.920 | 0.173 | 1.748 | 0.929 | 1.063 | 0.127 | 0.518 | 1.154 | 0.172 |
| 760 | 1.348 | 0.884 | 1.638 | 0.700 | 0.509 | 1.012 | 0.583 | 0.822 | 0.226 | 1.708 | 0.246 | 0.187 |
| 762 | 1.314 | 2.600 | 1.043 | 1.310 | 0.248 | 0.733 | 0.520 | 0.281 | 0.860 | 6.232 | 0.945 | 0.252 |
| 766 | 0.424 | 0.355 | 0.293 | 0.610 | 0.236 | 0.250 | 0.650 | 1.389 | 0.240 | 3.752 | 0.397 | 0.260 |
| 772 | 0.483 | 0.538 | 1.107 | 0.461 | 0.253 | 0.493 | 0.396 | 0.211 | 0.106 | 0.405 | 0.376 | 0.158 |
| 774 | 1.335 | 0.383 | 0.192 | 0.350 | 0.599 | 0.395 | 4.143 | 1.449 | 0.250 | 0.292 | 1.207 | 0.352 |
| 800 | 0.220 | 0.492 | 0.439 | 0.314 | 0.413 | 0.192 | 0.167 | 0.903 | 0.137 | 0.264 | 0.310 | 0.210 |
|  | | | | | | | | | | | | |
| *Sample Nō* | *IgG1 (µg/ml)* | | | | | | | | | | | |
| *714* | 0.030 | 0.295 | 0.014 | 0.014 | 0.037 | 0.035 | 0.064 | 0.138 | 0.024 | Not  assigned | 0.134 | 0.050 |
| *736* | 0.226 | 0.223 | 0.111 | 0.111 | 0.323 | 0.141 | 0.145 | 0.270 | 0.202 |  | 0.909 | 0.107 |
| *744* | 0.001 | 0.013 | <0.01 | 0.014 | <0.01 | <0.01 | 0.022 | <0.01 | 0.016 |  | 0.085 | 0.023 |
| *746* | 0.001 | 0.001 | <0.01 | <0.01 | 0.054 | <0.01 | 0.017 | <0.01 | <0.01 |  | <0.01 | <0.01 |
| *750* | 0.366 | 1.004 | 0.200 | 0.096 | 0.164 | 0.165 | 0.553 | 1.289 | 0.282 |  | 0.984 | 1.526 |
| *752* | 0.579 | 1.770 | 1.541 | 0.083 | 0.517 | 0.154 | 1.074 | 1.681 | 0.288 |  | 1.607 | 0.849 |
| *754* | 0.001 | 0.018 | 0.028 | <0.01 | 0.035 | 0.075 | 0.030 | 0.702 | <0.01 |  | 0.152 | 0.074 |
| *758* | 0.015 | 0.049 | <0.01 | 0.023 | 0.024 | 0.057 | 0.024 | 0.609 | 0.017 |  | 0.249 | 0.019 |
| *760* | 0.001 | 0.014 | <0.01 | <0.01 | <0.01 | 0.046 | <0.01 | <0.01 | <0.01 |  | 0.057 | <0.01 |
| *762* | 1.017 | 0.699 | 0.166 | 0.146 | 0.316 | 0.457 | 0.215 | 1.048 | 0.241 |  | 1.000 | 0.242 |
| *764* | 0.796 | 0.578 | 0.095 | 0.047 | 0.136 | 0.472 | 1.052 | 0.542 | 0.120 |  | 0.283 | 0.202 |
| *768* | 0.365 | 0.264 | 0.064 | 0.036 | 0.063 | 0.057 | 0.090 | 0.605 | 0.046 |  | 0.281 | 0.117 |
| *770* | 0.156 | 0.545 | 0.039 | 0.064 | 0.096 | 0.048 | 0.173 | 1.064 | 0.058 |  | 0.154 | 0.190 |
| *772* | 0.027 | 0.271 | 0.013 | 0.028 | 0.296 | 0.055 | 0.065 | 0.144 | 0.044 |  | 5.367 | 0.064 |
| *774* | 0.001 | 0.001 | <0.01 | <0.01 | 0.019 | <0.01 | 0.013 | <0.01 | <0.01 |  | <0.01 | <0.01 |
| *800* | 4.084 | 1.506 | 0.123 | 0.123 | 0.211 | 0.861 | 0.324 | 1.474 | 0.320 |  | 6.357 | 1.492 |
|  | | | | | | | | | | | | |
| *Sample Nō* | *IgG2 (µg/ml)* | | | | | | | | | | | |
| *714* | 0.436 | 0.371 | 0.293 | 1.709 | 0.709 | 0.497 | 1.780 | 0.406 | 0.594 | Not  assigned | 1.202 | 0.208 |
| *736* | 3.123 | 0.770 | 1.037 | 3.664 | 1.295 | 3.856 | 2.401 | 31.474 | 1.571 |  | 1.470 | 0.534 |
| *744* | 2.728 | 1.381 | 2.381 | 10.465 | 13.333 | 7.850 | 4.518 | 3.857 | 7.476 |  | 4.800 | 2.392 |
| *746* | 6.750 | 0.856 | 1.353 | 2.366 | 5.718 | 1.306 | 5.697 | 6.870 | 1.149 |  | 3.782 | 1.078 |
| *750* | 3.122 | 1.297 | 17.341 | 10.784 | 4.997 | 6.946 | 4.967 | 25.539 | 3.230 |  | 4.694 | 35.457 |
| *752* | 1.725 | 1.384 | 6.349 | 2.697 | 5.806 | 31.647 | 13.748 | 17.268 | 7.404 |  | 23.680 | 6.022 |
| *754* | 2.695 | 1.245 | 10.704 | 5.698 | 3.925 | 24.288 | 14.816 | 74.022 | 3.938 |  | 10.218 | 8.261 |
| *758* | 7.485 | 2.024 | 3.322 | 28.982 | 11.560 | 10.876 | 10.443 | 48.215 | 14.078 |  | 8.093 | 17.168 |
| *760* | 3.468 | 2.892 | 3.374 | 8.039 | 2.156 | 21.112 | 2.646 | 9.616 | 1.837 |  | 6.197 | 1.107 |
| *762* | 6.875 | 2.533 | 0.786 | 2.078 | 0.749 | 3.088 | 1.718 | 3.542 | 0.989 |  | 2.755 | 0.630 |
| *764* | 8.108 | 2.339 | 2.939 | 14.383 | 19.347 | 1.822 | 4.895 | 14.075 | 5.269 |  | 8.294 | 14.415 |
| *768* | 7.159 | 0.492 | 0.952 | 3.054 | 1.942 | 1.504 | 5.850 | 10.011 | 1.536 |  | 2.441 | 0.934 |
| *770* | 1.999 | 1.336 | 4.120 | 5.089 | 6.307 | 4.782 | 3.968 | 42.625 | 3.786 |  | 4.325 | 9.848 |
| *772* | 3.568 | 1.305 | 2.254 | 9.616 | 4.331 | 13.215 | 7.445 | 1.136 | 2.145 |  | 2.927 | 1.391 |
| *774* | 0.479 | 0.238 | 0.296 | 1.831 | 0.762 | 0.896 | 0.967 | 0.827 | 0.329 |  | 0.319 | 0.061 |
| *800* | 1.155 | 1.027 | 1.020 | 1.699 | 1.202 | 1.865 | 1.462 | 3.799 | 1.086 |  | 1.484 | 1.510 |
